# Dynamics of platelet activation in cold-stored low-titer group O whole blood over 8 days: insights relative to standard platelet concentrates

**DOI:** 10.1186/s40635-026-00945-x

**Published:** 2026-07-13

**Authors:** Fabrice Cognasse, Karine Desseux, Véronique Artero, Véronique Duraffourg, Charles-Antoine Arthaud, Amélie Prier, Marie-Ange Eyraud, Victorine Maillot, Damien Crespe, Christophe Martinaud, Sylvain Ausset, Pierre Tiberghien, Laurent Aoustin, Patricia Chavarin, Sébastien Banzet, Nadira Frescaline, Hind Hamzeh-Cognasse

**Affiliations:** 1 Établissement Français du Sang Auvergne-Rhône-Alpes and INSERM U1059, Université Jean Monnet, 25 Boulevard Pasteur, Saint-Étienne, 42100 France; 2 INSERM U1059 SAINBIOSE, Université Jean Monnet, Mines Saint-Étienne, Saint-Étienne, France; 3 Centre de Transfusion Sanguine des Armées, Clamart, France; 4 French Military Medical Service Academy, École du Val-de-Grâce, Paris, France; 5 Établissement Français du Sang, La Plaine Saint-Denis, France; 6 UMR RIGHT 1098, Inserm, EFS, Université de Franche-Comté, Besançon, France

**Keywords:** Platelets, Whole blood, Inflammation, LTOWB, Cold storage, Flow cytometry

## Abstract

**Background:**

Low-titer group O whole blood (LTOWB) is increasingly used for hemorrhagic resuscitation in trauma and critical care, but the phenotype of platelets maintained within refrigerated LTOWB remains incompletely characterized. Because platelet activation may influence not only hemostatic competence but also transfusion-related inflammatory and pulmonary responses in critically ill patients, we assessed platelet surface markers in LTOWB in comparison with standard platelet concentrates and examined their evolution during 8 days of cold storage.

**Methods:**

LTOWB units prepared from qualified group O donors were stored under refrigerated conditions and studied at day 0 and day 8. Platelet phenotype was assessed by flow cytometry using CD41, CD62P, and CD63 under basal conditions and after thrombin receptor-activating peptide (TRAP) stimulation. LTOWB was compared with apheresis platelet concentrates (APC) and buffy coat-derived platelet concentrates (BC-PC).

**Results:**

Compared with APC and BC-PC, LTOWB showed a broader distribution of CD41-positive events and a modestly lower median CD41 signal. Basal CD62P in LTOWB was lower than in APC but higher than in BC-PC, whereas basal CD63 in LTOWB was higher than in both comparator products. Between day 0 and day 8, basal CD62P and CD63 increased in LTOWB. TRAP induced marked upregulation of CD62P and CD63 at both time points; stimulated CD63 was lower at day 8 than at day 0, whereas stimulated CD62P remained high.

**Conclusion:**

Cold-stored LTOWB displays a distinct platelet activation phenotype characterized by progressive basal activation during refrigerated storage together with persistent agonist-inducible responses through day 8. These findings justify further functional and translational studies integrating platelet, endothelial, and lung-injury readouts to determine whether this storage-associated phenotype has consequences for microvascular hemostasis, immunothrombosis, or transfusion-associated pulmonary complications after hemorrhagic resuscitation.

## Introduction

Whole blood has re-emerged as a pragmatic resuscitation product because it delivers red cells, plasma, and platelets in a single unit while simplifying logistics in prehospital and hospital settings. Civilian transfusion practice has increasingly incorporated low-titer group O whole blood (LTOWB), particularly for early hemorrhage control, and recent clinical series support its operational feasibility and potential outcome benefit in selected bleeding populations [1–3]. This question is particularly relevant in critically ill bleeding patients, in whom transfused blood products interact with a primed endothelium and an activated innate immune system. In this setting, storage-associated platelet activation may affect not only clot formation but also platelet–endothelial and platelet–leukocyte crosstalk, including pathways potentially relevant to pulmonary transfusion complications. Defining the platelet phenotype in LTOWB is therefore important not only for hemostatic interpretation, but also for a broader assessment of its biological safety profile.

At the same time, platelet behavior within cold-stored whole blood remains incompletely defined. Processing steps such as platelet-sparing leukoreduction, refrigerated storage, and delayed component preparation may alter platelet activation state without fully abolishing agonist responsiveness [4–9]. These issues are clinically relevant because the hemostatic contribution of LTOWB depends not only on platelet quantity but also on the biological state of platelets at the time of transfusion [7–11].

Platelets also participate in thromboinflammatory signaling, making phenotypic characterization informative beyond simple cell counting [12, 13]. The present study was therefore deliberately restricted to platelet analyses. We compared basal expression of CD41, CD62P, and CD63 in LTOWB with that observed in apheresis platelet concentrates (APC) and buffy coat-derived platelet concentrates (BC-PC), and we assessed the evolution of these markers in LTOWB from day 0 to day 8 under basal conditions and after TRAP stimulation.

## Materials and methods

**Fig. 1:**
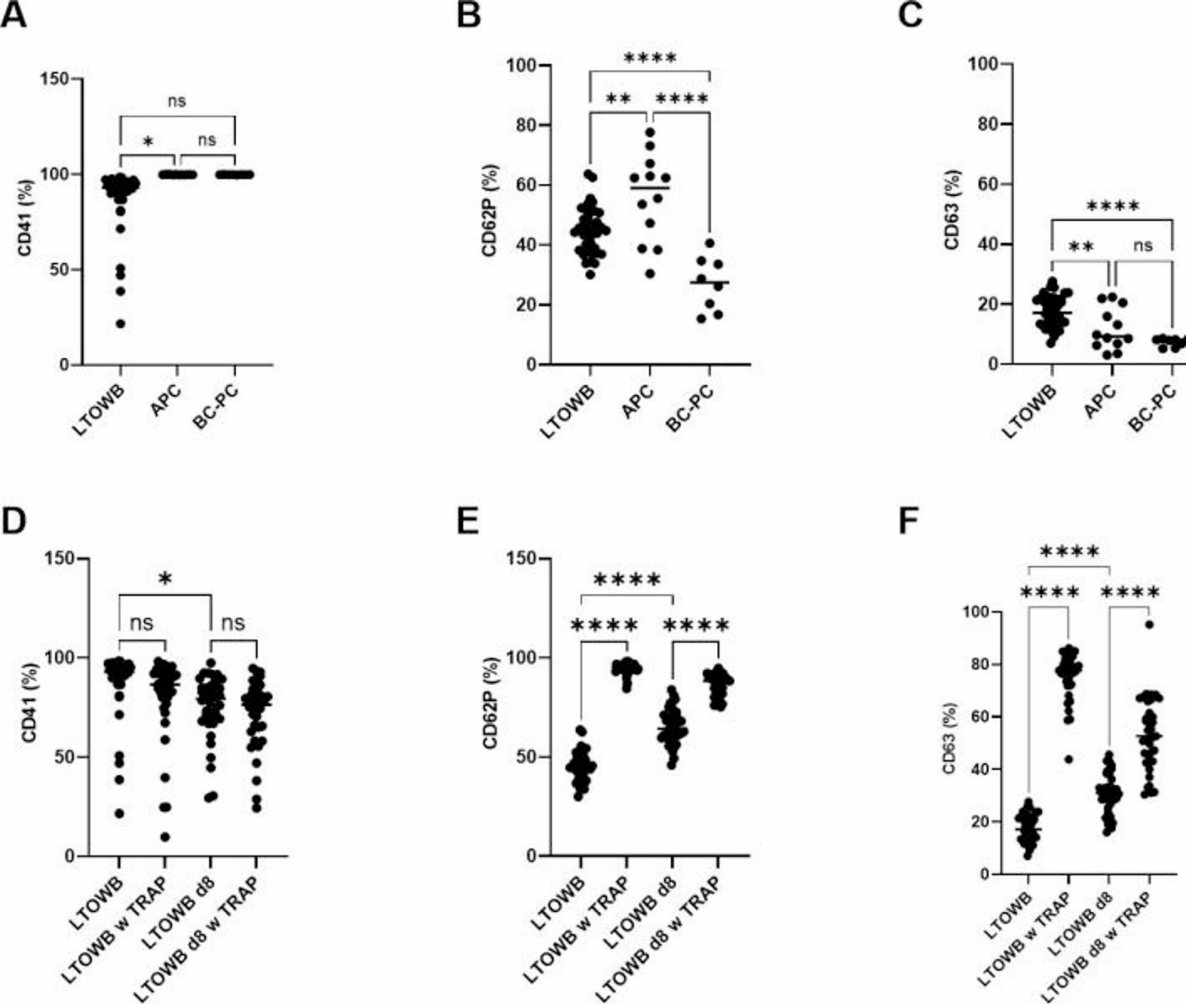
Platelet phenotyping in low-titer group O whole blood (LTOWB) compared with conventional platelet concentrates and during 8-day cold storage. Panels **A**–**C** show basal expression of CD41, CD62P, and CD63 in LTOWB, apheresis platelet concentrates (APC), and buffy coat-derived platelet concentrates (BC-PC). Panels **D**–**F** show expression of CD41, CD62P, and CD63 in LTOWB at day 0 and day 8 under basal conditions and after TRAP-6 stimulation. Each dot represents one measurement from an individual blood product unit in the indicated group and condition; technical replicates are not shown. Horizontal bars indicate medians. Exact group sizes were LTOWB basal comparison (*n* = 38), APC (*n* = 12), BC-PC (*n* = 8), LTOWB day 0 (*n* = 38), and LTOWB day 8 (*n* = 38). APC and BC-PC were analyzed during standard room-temperature storage at 20–24 °C. LTOWB was stored at 2–6 °C and stimulated with TRAP-6 (50 µg/mL, 30 min) where indicated. Panels A-C: cross-product basal comparisons were analyzed using one-way ANOVA followed by Tukey correction. Panels D-F: LTOWB day 0/day 8 basal and TRAP-6-stimulated conditions were analyzed using Friedman test followed by Dunn’s multiple-comparisons test. *p < 0.05, **p < 0.01, ****p < 0.0001; ns, not significant

This study focuses exclusively on platelet analyses corresponding to Fig. 1. LTOWB units were prepared from standard whole blood donations obtained from qualified group O donors selected according to institutional criteria already used in the parent study [14]. Donations were processed within 12 h after collection, leukoreduced using a platelet-sparing strategy, and stored under refrigerated conditions at 2–6 °C. In this platelet-focused version, LTOWB samples were analyzed at day 0 and day 8. APC and BC-PC were produced within the French Blood Establishment and analyzed at 20–24 °C. Immediately before sampling, each blood product was gently mixed according to local blood-bank procedures to obtain a homogeneous suspension while avoiding vigorous agitation. Samples were collected aseptically from LTOWB, APC, or BC-PC units and processed without intentional delay. Platelet suspensions were adjusted to 300,000 platelets/µL. For TRAP-6 conditions, samples were incubated with TRAP-SFFLRN peptide at a final concentration of 50 µg/mL for 30 min at room temperature before antibody staining [15, 16]. Samples were centrifuged at 2,500 g for 5 min at room temperature. This analytical centrifugation step was applied consistently to all product types. Samples were then labeled for 30 min at room temperature in the dark with anti-CD41 (HIP-8 APC; BD Biosciences, 559777), anti-CD62P (AK-4 PE; BD Biosciences, 555524), and anti-CD63 (H5C6 FITC; BD Biosciences, 557288). Labeled cells were analyzed on a Cytek Northern Light flow cytometer. Instrument settings, compensation, and gating strategy were kept constant across conditions. The analytical gate used to define platelet-associated events was based on forward- and side-scatter properties combined with CD41 expression, with exclusion of debris and doublets.

CD41 was used to identify platelet-associated events. CD41 corresponds to glycoprotein IIb/integrin alpha-IIb, part of the alpha-IIb beta-3 complex constitutively expressed on the platelet surface. CD62P corresponds to P-selectin, an alpha-granule protein with low surface expression on resting platelets and rapid surface exposure after alpha-granule secretion. CD63 is a tetraspanin/lysosomal- and dense-granule-associated marker with low surface expression on resting platelets and increased surface exposure after degranulation/advanced activation. TRAP-6 activates the thrombin receptor PAR-1 and was used as a strong agonist expected to induce platelet granule secretion and increased CD62P/CD63 expression. In this study, TRAP-6 stimulation was used as an ex vivo responsiveness readout and not as a stand-alone test of clinical hemostatic efficacy [17–20]. The number of units analyzed was as follows: LTOWB in panels A-C, *n* = 38; APC, *n* = 12; BC-PC, *n* = 8; LTOWB day 0 in panels D-F, *n* = 38; and LTOWB day 8 in panels D-F, *n* = 38.

Data are displayed as individual values with medians. Cross-product basal comparisons were analyzed using one-way ANOVA followed by Tukey correction. LTOWB day 0/day 8 basal and TRAP-6-stimulated conditions were analyzed using a Friedman test, followed by Dunn’s multiple-comparisons test. Adjusted two-sided p values less than 0.05 were considered statistically significant. Exact p-value annotations in the figure correspond to corrected post-hoc comparisons.

## Results

The proportion of CD41-positive events was tightly clustered near 100% in APC and BC-PC, whereas LTOWB displayed a broader distribution with several lower values and a modestly lower median (Fig. 1A). Because CD41 was used here as an identification marker within the analytical gate, this broader distribution should be interpreted as heterogeneity of platelet-associated events or signal distribution rather than as a direct platelet count or proof of reduced platelet number. This pattern therefore indicates greater event heterogeneity in LTOWB than in standard platelet concentrates, without by itself establishing impaired platelet function.

Basal CD62P differed across products (Fig. 1B). APC showed the highest expression, BC-PC the lowest, and LTOWB an intermediate phenotype. Thus, basal CD62P in LTOWB was lower than in APC but higher than in BC-PC. Basal CD63 followed a different pattern (Fig. 1C): LTOWB showed the highest values, APC intermediate values, and BC-PC the lowest values. Taken together, these data indicate that LTOWB platelets cannot be described simply as globally more or less activated than conventional platelet concentrates; rather, the pattern depends on the marker examined.

Within LTOWB, CD41-positive events remained predominant at both time points and were not materially altered by TRAP stimulation (Fig. 1D). However, basal CD41 at day 8 was modestly lower than basal CD41 at day 0, consistent with storage-related heterogeneity of platelet-associated events during refrigerated storage.

Basal CD62P increased from day 0 to day 8 (Fig. 1E), indicating progressive storage-associated activation. TRAP induced a marked rise in CD62P at both time points, and stimulated values remained high at day 8. Basal CD63 also increased between day 0 and day 8 (Fig. 1F). TRAP markedly increased CD63 expression at both time points, but stimulated CD63 at day 8 was lower than stimulated CD63 at day 0, consistent with partial attenuation of agonist-inducible degranulation over time. Overall, Fig. 1 supports two concurrent phenomena in LTOWB: increasing basal activation during storage and persistence of inducible responses through day 8.

## Discussion

This study addresses a single question: the platelet phenotype of cold-stored LTOWB and its short-term evolution during refrigerated storage. The data show that LTOWB differs from APC and BC-PC at baseline, with broader CD41 event distribution, intermediate basal CD62P, and higher basal CD63. They also show that storage from day 0 to day 8 is accompanied by rising basal activation while TRAP-inducible responses remain detectable. From a critical care perspective, this phenotype may matter beyond ex vivo platelet description alone. In massively bleeding patients, transfused LTOWB is delivered into a host environment characterized by endothelial activation, systemic inflammation, and a heightened risk of immunothrombotic and pulmonary complications. In such a context, storage-associated platelet activation may influence not only primary hemostasis, but also platelet–endothelial and platelet–leukocyte interactions, making phenotypic assessment relevant to both efficacy and biological safety.

The present study demonstrates that cold-stored LTOWB has a distinct ex vivo platelet phenotype compared with conventional platelet concentrates. This phenotype should not be interpreted as simply “more activated” or “less functional”, because the pattern depended on the marker examined [4–11]. Basal CD62P, a marker of alpha-granule secretion, was intermediate in LTOWB, whereas basal CD63, a marker of degranulation/advanced activation, was higher than in both comparator products. This divergence suggests that different compartments of platelet activation may be differentially affected by product environment, processing history, and storage temperature [7–11, 21].

The clinical significance of increased basal CD62P/CD63 cannot be determined from the present data alone. It may reflect beneficial hemostatic priming, a component of the platelet storage lesion, proinflammatory potential, or a laboratory phenotype without direct clinical relevance. Mechanistically, surface P-selectin may support platelet-leukocyte and platelet-endothelial interactions, whereas CD63 upregulation may reflect deeper granule mobilization and storage-associated remodeling. These pathways are relevant to thromboinflammation, microvascular interactions, and potentially pulmonary transfusion biology. However, the present study did not assess platelet adhesion under flow, aggregation, clot formation, thrombin generation, platelet-leukocyte aggregates, endothelial activation, complement activation, lung-injury readouts, or patient outcomes.

Accordingly, no direct conclusion can be drawn from these data regarding bleeding control, clinical hemostatic efficacy, microvascular thrombosis, ARDS/TRALI risk, or survival after LTOWB transfusion. The maintained TRAP-6-induced CD62P and CD63 responses indicate residual agonist-inducible signaling through day 8, while the lower stimulated CD63 response at day 8 may indicate partial attenuation of secretory reserve. This ex vivo phenotype is therefore hypothesis-generating and requires dedicated functional and translational studies before clinical implications can be established.

This study has several limitations. First, it relies on a restricted flow-cytometric marker panel and does not include complementary functional assays such as light transmission aggregometry, whole-blood aggregometry, viscoelastic testing, thrombin generation, platelet adhesion under flow, microparticle analysis, or in vivo recovery studies. Second, APC and BC-PC were included as basal reference comparator products and were not analyzed as matched day 0/day 8 cold-storage controls; therefore, the study does not provide a definitive cross-product functional ranking. Third, TRAP-6 stimulation was performed to assess LTOWB responsiveness over storage but was not performed in APC or BC-PC under matched storage conditions. Fourth, this study is an ancillary ex vivo platelet-phenotyping analysis focused exclusively on platelet markers in LTOWB and comparator platelet components. LTOWB was the primary product of interest. All available LTOWB units with valid flow-cytometry data were included (*n* = 38). APC (*n* = 12) and BC-PC (*n* = 8) were included as reference platelet-component comparators to contextualize the basal LTOWB phenotype, rather than as fully powered or storage-age-matched comparator groups. Because the platelet-phenotyping endpoint was exploratory and ancillary to the parent LTOWB program, no a priori sample-size calculation was performed. This point is now acknowledged as a limitation.

Finally, these ex vivo phenotypic data should not be extrapolated directly to clinical hemostatic efficacy, thrombosis, ARDS/TRALI, or patient outcomes. No parallel extended product-quality panel was available for the same units. Therefore, electrolyte levels, LDH, pH, glucose/lactate, hemolysis, platelet content over storage, and microparticle content could not be integrated into the present analysis. The CD62P/CD63 phenotype should consequently be interpreted as a focused platelet activation readout rather than as a comprehensive assessment of LTOWB product quality.

In conclusion, cold-stored LTOWB exhibits a distinct ex vivo platelet activation phenotype characterized by progressive basal CD62P/CD63 expression during 8 days of refrigerated storage and persistent TRAP-6-inducible responses. These data support further investigation of LTOWB platelet competence with complementary functional, endothelial, leukocyte, and lung-injury assays, but they do not establish clinical hemostatic efficacy or safety outcomes.

## Data Availability

The data presented in this study are available from the corresponding author on reasonable request, subject to legal restrictions.
